# Host-Toxin Interactions Involving EspC and Pet, Two Serine Protease Autotransporters of the Enterobacteriaceae

**DOI:** 10.3390/toxins2051134

**Published:** 2010-05-14

**Authors:** Fernando Navarro-Garcia, Michael Sonnested, Ken Teter

**Affiliations:** 1 Department of Cell Biology, Centro de Investigación y de Estudios Avanzados del IPN (CINVESTAV-Zacatenco), Ap. Postal 14-740, 07000 México DF, Mexico; Email: msonnested@cell.cinvestav.mx; 2 Burnett School of Biomedical Sciences, College of Medicine, University of Central Florida, 12722 Research Parkway, Orlando, FL 32826, USA; Email: kteter@mail.ucf.edu

**Keywords:** :enterotoxins, autotransporter proteins, actin cytoskeleton, diarrheagenic *E. *coli, serine proteases

## Abstract

EspC and Pet are toxins secreted by the diarrheagenic enteropathogenic and enteroaggregative *Escherichia coli *pathotypes, respectively. Both toxins have a molecular mass around 110 kDa and belong to the same protein family called Serine Protease Autotransporters of the *Enterobacteriaceae* (SPATE). Furthermore, both toxins act within the cytosol of intoxicated epithelial cells to disrupt the architecture of the actin cytoskeleton. This cytopathic and enterotoxic effect results from toxin cleavage of the actin-binding protein fodrin, although the two toxins recognize different cleavage sites on fodrin. EspC and Pet also have dramatically different mechanisms of entering the target cell which appear dependent upon the *E. coli *pathotype. In this review, we compare/contrast EspC and Pet in regards to their mode of delivery into the target cell, their effects on fodrin and the actin cytoskeleton, and their possible effects on the physiology of the intestinal epithelial cell.

## 1. Introduction

Diarrheagenic *Escherichia coli* have been loosely classified into six major categories based on clinical associations, serotyping, phenotypic assays, and identification of virulence characteristics. The six pathotypes are enteroaggregative *E. coli* (EAEC), enteropathogenic *E. coli *(EPEC), enterotoxigenic *E. coli* (ETEC), enterohemorragic *E. coli *(EHEC), enteroinvasive *E. coli *(EIEC), and diffusely adherent *E. coli *(DAEC) [1,2]. EAEC and EPEC each produce a distinct serine protease enterotoxin: EAEC secretes plasmid-encoded toxin (Pet), while EPEC releases E. coli secreted protein C (EspC). Pet and EspC are both Serine Protease Autotransporters of the *Enterobacteriaceae* (SPATEs) that generate diarrheagenic effects through alterations of the actin cytoskeleton. Despite these similarities, Pet and EspC exhibit distinct host-toxin interactions and may play different roles in pathogenesis.

## 2. EAEC and EPEC Infections

EAEC infections occur worldwide in both developed and developing countries [3,4]. Children and adults living in developed countries are susceptible to acute infection with EAEC, as are travelers visiting developing countries. Persistent EAEC infections occur in HIV-infected individuals and children from developing countries. Mucosal damage resulting from EAEC infection has been detected in patients, animal models, and *in vitro* organ culture [5,6,7,8,9,10].

A defining feature of EAEC is its aggregative or “stacked brick” pattern of adherence to intestinal tissue and cultured epithelial cells [9,10,11]. This aggregative adherence (AA) phenotype is derived from the ability of EAEC to attach to other EAEC, to epithelial cells, and to the surface of tissue culture plates. Adherence involves AA fimbriae expressed from a 65-MDa plasmid (pAA) [12,13]. Upon adhesion to the epithelium, EAEC forms a biofilm and induces the production of a thick mucus layer over the intestinal surface. The release of toxins into the gut further contributes to enterotoxicity [3,4]. One such toxin is Pet, a SPATE encoded on the pAA plasmid [14]. 

EPEC is a leading cause of acute infantile diarrhea in developing countries [1]. The pathogen adheres to epithelial cells on an actin-rich pedestal, which replaces the intestinal microvilli. This structure, known as the attaching and effacing (A/E) lesion, reduces the absorptive capacity of the intestine and can be detected in patients, animal models, and cell culture [15,16,17,18,19]. The A/E lesion results from the injection of bacterial effector proteins into the host cell cytosol through a type III secretion system [20]. Effector proteins, as well as the type III secretion apparatus, are encoded within a 35.6kilobase (kb) pathogenicity island termed the locus of enterocyte effacement (LEE) [21,22]. A second chromosomal pathogenicity island encodes EspC, a SPATE enterotoxin released by the type V secretion system [23,24]. Ler, a transcriptional activator encoded in the LEE, couples the expression of EspC with the expression of LEE virulence factors [25,26]. 

## 3. Structure of Pet and EspC

**Figure 1:**
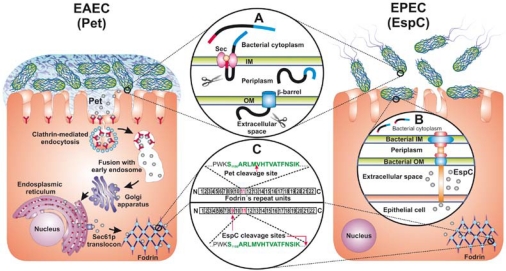
Structure, secretion mechanism, cytoplasm translocation and interaction with the protein target of Pet and EspC. (Circle A) Structure and secretion of Pet and EspC through the type V secretion system (T5SS). The left cell shows Pet endocytosis and retrograde trafficking to reach its intracellular target. The right cell shows EspC translocation through the cooperation of the T5SS and the type III secretion system (circle B). (Circle C) Different cleavage sites for Pet and EspC on fodrin, a common intracellular target.

Pet and EspC belong to the SPATE subfamily of autotransporters [27,28]. SPATEs and other autotransporters use a type V secretion system for export to the extracellular space [29,30]. This process is thought to be a self-contained event in which the amino acid sequence of the autotransporter contains all of the information necessary for passage through the inner membrane as well as the outer membrane. To mediate its own secretion, an autotransporter contains three functional domains: an *N*-terminal leader sequence, an extracellular passenger domain, and a *C*-terminal β-barrel domain. The *N*-terminal leader sequence initiates Sec-dependent transport across the inner membrane and is proteolytically removed in the periplasmic space. The *C*-terminal domain forms a β-barrel pore in the outer membrane which facilitates delivery of the passenger domain to the extracellular space. The passenger domains of some autotransporters remain anchored to the extracellular face of the outer membrane, but SPATEs are released from the bacterial cell by proteolytic nicking of a site between the β-barrel pore and the passenger domain (Figure 1). The mature, secreted SPATEs are 104-110 kDa toxins that contain a typical N-terminal serine protease catalytic domain followed by a highlyconserved β-helix motif, which is present in nearly all autotransporters [27,28,29,30]. Although the general process of SPATE secretion is understood, the details of many events in SPATE biogenesis (chaperone function in the periplasm; mechanism of β-barrel insertion into the outer membrane; translocation pathway across the outer membrane; proteolytic release of the mature protein from the outer membrane; *etc*.) remain unresolved. 

Pet and EspC share a similar structural organization. *In silico* analysis indicated that the Pet leader sequence is cleaved between residues A^52^ and A^53^, while the cleavage site linking the β-domain to the mature Pet protein occurs between residues N^1018^ and N^1019^[14]. The secreted Pet passenger domain is thus a 104 kDa toxin that encompasses amino acid residues 53 to 1018 of nascent Pet. In the case of EspC, the leader sequence extends from amino acids 1 to 53; the 110 kDa passenger domain extends from amino acids 54 to 1,030; and the β-barrel pore extends from amino acids 1,031 to 1,306 [31]. The serine protease motifs of Pet (GDSGS^260^G) and EspC (GDSGS^256^G) are identical but differ slightly regarding the location of the active site serine residue within the passenger domain. Although some secreted autotransporters mediate their own proteolytic release from the bacterial surface, neither Pet nor EspC required a functional serine protease motif for secretion [23,32]. The proteolytic event which releases both toxins is likely mediated by an outer membrane protease or proteases. However, the protease(s) responsible for release from the outer membrane has not yet been identified. Identification and characterization of the responsible protease(s) would significantly advance our understanding of the type V secretion system for SPATE proteins.

Interestingly, correct folding of the Pet passenger domain appears to require a currently unidentified host-specific accessory factor [33]. A substantial fraction of Pet secreted from a HB101 lab strain of *E. coli* transformed with the minimal Pet clone pCEFN1 [14] was detected in a misfolded, inactive conformation. The misfolded variant of Pet was not produced by EAEC and did not result from protein overproduction in the lab strain of *E. coli*. This report was consistent with a developing model of autotransporter biogenesis in which accessory factors are necessary for the folding and/or secretion of an autotransporter [34,35,36,37,38]. The folding efficiency of EspC has not yet been examined, but the extended regions of SPATE surface hydrophobicity predicted from computer modeling suggests that efficient, proper folding of EspC and other SPATEs may also require host accessory factors [33]. These factors could further assist SPATE biogenesis by (i) facilitating β-barrel insertion into the outer membrane; (ii) maintaining the passenger domain in a translocation-competent state for movement across the outer membrane; and (iii) protecting the nascent passenger domain from degradation by periplasmic proteases. The emerging role of chaperones and other accessory factors in SPATE biogenesis calls into question the "autotransporter" label for this family of proteins. However, the realization that chaperone-mediated events function in autotransporter secretion has consequently identified new pharmacological targets to possibly block the release of virulence factors by the type V secretion system. 

In addition to Pet and EspC, SPATE family members include SigAand SepA from *Shigella flexneri*, Sat from DAEC and uropathogenic *E. coli*, Pic from EAEC and *Shigella* strains, EspP from EHEC, and Tsh from avian pathogenic *E. coli* [27,28]. Phylogenetic analysis of the serine protease regions which comprise the N-terminal third of SPATE passenger domains has divided SPATE family members into two classes [39]. Class I SPATEs are all cytotoxic to epithelial cells. These toxins include Pet, EspC, EspP, SigA, and Sat. Class II SPATEs are more diverse with regard to toxin function, although several are known to cleave mucin. Pic, for example, is a chromosomally-encoded mucinase [40] that may promote intestinal colonization by an unknown mechanism [41]. Besides Pic, Class II SPATEs include SepA and Tsh. Phylogenetic analysis has indicated that Pet, EspC, and Sat are more closely related to each other than any other known SPATE [39]. Pet and EspC share a 55% amino acid identity and 70% amino acid similarity overall; within the passenger domain, there is 49% amino acid identity [14]. Despite the relatedness of SPATE family members, the activity of SPATEs is diverse and each one of them needs to be characterized as an individual virulence factor in the context of each SPATE-producing pathogen. As discussed below, even two closely related SPATEs such as Pet and EspC are distinct in many aspects.

## 4. Pet and EspC Activity on Epithelial Cells

Pet was originally identified as an immunogenic protein recognized by antisera from children infected by EAEC [8]. Due to the diarrheagenic nature of EAEC, a potential role for Pet as an enterotoxin was investigated. Pet was shown to increase short-circuit currents (Isc) and decrease electrical resistance of rat jejunum mounted in Ussing chambers [42]. These effects were accompanied by mucosal damage, exfoliation of cells, and development of crypt abscesses. Similar effects had been previously observed for *in vitro* organ culture [9] and T84 cultured epithelial cells [6] exposed to the EAEC bacterium. Deletion of the *pet *gene was reported to lessen the pathogen-induced effects in terms of exfoliation and widening of crypt openings [43]. 

Epithelial cells exposed to 25 μg/mL of purified Pet begin to display phenotypic changes after two hours of toxin exposure [32]. A 10 minute toxin exposure to 10 μg/mL of Pet followed by a five hour incubation in the absence of toxin was sufficient for productive intoxication. The time- and dose-dependent cytopathic effects of Pet, which could be detected by light microscopy, were characterized by an initial cell elongation followed by cell rounding and, ultimately, detachment from the substratum. Cell rounding and release from the substratum resulted from contraction of the cytoskeleton, loss of actin stress fibers, and release of focal contacts. Pet disruption of the actin cytoskeleton required a functional serine protease motif, as its cytopathic activity was inhibited by the serine protease inhibitor phenylmethylsulfonyl fluoride (PMSF). Likewise, an S260I active site mutation eliminated the *in vitro* protease activity and *in vivo* cytopathic activity of Pet. Collectively, these observations indicated that the serine protease activity of Pet is responsible for alterations to the actin cytoskeleton which generate cytopathic and enterotoxic effects. 

EspC was originally isolated from the supernatant of EPEC grown in the presence of epithelial cells [44]. When applied to rat jejunal segments mounted in Ussing chambers, EspC generated enterotoxic effects that were manifested as rises in short-circuit currents and transepithelial electrical potential differences [24]. Yet, in contrast to the demonstrated role of Pet in EAEC pathogenesis, deletion of the *espC* gene did not affect any obvious aspect of EPEC pathogenesis such as formation of the A/E lesion [23]. Purified EspC also generated cytopathic effects against cultured epithelial cells [45]. Like Pet, EspC-induced cell damage resulted from serine protease activity: no cytoskeletal alterations were observed in cells co-incubated with EspC and PMSF or in cells exposed to an EspC mutant containing an inactivating S256I mutation in the serine protease active site. 

EspC and Pet elicit similar cytopathic effects from cultured cells: both toxins induce contraction of the actin cytoskeleton, cell rounding, and detachment from the substratum [32,45]. Internalization into the host cell was required for both EspC and Pet to cause their cytopathic effects [45,46]. However, the cytoskeletal alterations resulting from EspC intoxication were only evident when the toxin was applied at a concentration of 120 μg/mL for at least six hours [45]. Pet, in comparison, was effective at lower toxin concentrations and after much shorter incubation periods [32,45]. These differences occurred even though EspC and Pet share the same serine protease motif and exhibit a 49% amino acid identity in the passenger domain [14]. 

## 5. Delivery of Pet and EspC to the Host Cell Cytosol

The greater potency of purified Pet in comparison to purified EspC is due to the distinct mechanisms of toxin delivery to the host cell. Pet is internalized by receptor-mediated endocytosis [47] whereas EspC is internalized by a different mechanism that requires EPEC contact with the host cell and production of a type III secretion system [31,48]. Thus, in contrast to Pet toxicity assays, the experimental model of cell intoxication with purified EspC does not represent a physiological internalization mechanism.

Pet must be internalized to induce a cytopathic effect [46]. The toxin binds to the epithelial plasma membrane at 4 °C but only damages cells after the temperature is raised to 37 °C for at least two hours [32]. Endocytosis is blocked at 4 °C, so the temperature requirement for productive intoxication indicated that Pet does not act at the cell surface. Binding to the plasma membrane at 4 °C further suggested that a possible specific surface receptor is recognized by Pet, although this receptor has yet to be identified. Further evidence for the functional role of endocytosis in Pet intoxication was provided by drug- and siRNA-induced disruptions to clathrin-dependent endocytic events [47]. These treatments effectively blocked Pet activity against cultured cells. In contrast, neither drug-induced disruptions to clathrin-independent endocytosis nor siRNA knockdown of caveolae-dependent endocytosis inhibited Pet activity against cultured cells. Collectively, these observations mapped a clathrin-dependent endocytic route for Pet internalization (Figure 1).

The two hour time lag between toxin exposure and toxin damage further indicated that multiple intracellular transport steps were required for productive Pet intoxication. Indeed, the vectorial transport of Pet from the cell surface to the endosomes, from the endosomes to the Golgi apparatus, and from the Golgi apparatus to the endoplasmic reticulum (ER) was recorded by confocal microscopy [49]. The functional importance of this trafficking pattern was demonstrated with brefeldin A, a drug that blocks transport from the endosomes to the Golgi apparatus: cells exposed to brefeldin A were resistant to Pet [46]. In the ER, Pet associated with the Sec61p translocon pore before entering the cytosol as an intact 104 kDa protein [49]. This translocation event involved the mechanism of ER-associated degradation (ERAD), as a subset of mutant CHO cells with aberrant ERAD activity were highly resistant to Pet [49].

Many AB toxins also exploit ERAD to enter the target cell cytosol [50]. For these toxins, the catalytic A subunit dissociates from the cell-binding B subunit in the ER. The dissociated A subunit then unfolds and consequently activates ERAD, an endogenous quality control system which removes misfolded proteins from the ER by exporting them to the cytosol through Sec61p and/or Derlin-1 protein-conducting channels [51]. ERAD substrates are degraded in the cytosol by the ubiquitin-proteasome pathway, but the A subunits of ER-translocating toxins lack lysine residues for ubiquitin conjugation and thus persist in the cytosol [52]. Pet, the only non-AB toxin known to use the ERAD translocation mechanism, does not exhibit the arginine-over-lysine amino acid bias seen in the A subunits of ER-translocating toxins [14]. Furthermore, Pet activation of the ERAD system does not appear to involve substantial unfolding of the toxin [53]. Whereas toxin A chains actually unfold to trigger their ERAD-mediated translocation [54,55,56,57], Pet maintains a folded conformation and instead masquerades as a misfolded protein for ERAD processing [53]. The partial solvent exposure of aromatic amino acid residues in the native structure of Pet may facilitate its identification as a misfolded protein. The recognition of Pet as a misfolded protein was further highlighted by the activation of another ER quality control system, the unfolded protein response, in cells exposed to Pet or the inactive S260I Pet mutant [53]. 

Pet travels from the cell surface to the ER before exploiting ERAD to enter the cytosol where it damages the actin cytoskeleton (Figure 1). These events have been deduced from research using cell culture systems and purified Pet. Recent work using EAEC-treated culture cells has added additional, physiological detail to the intoxication process [58]. Pet secretion by EAEC during infection was inhibited at the transcriptional level by eukaryotic cell culture medium but not by the same medium supplemented with tryptone. In the presence of tryptone, EAEC released enough Pet to cause a toxic effect. However, toxicity was not observed when cultured cells were exposed to a HB101 lab strain of *E. coli *that was engineered to secrete high levels of Pet. Unlike EAEC, HB101 did not adhere to the cultured cells. A ∆*pet* EAEC strain that could adhere to the epithelial surface lacked toxicity as well. These collective observations indicated that Pet-induced toxicity requires an appropriate environment, as mimicked by tryptone-containing medium, and bacterial adhesion to the target cells. Enhanced Pet secretion in the presence of tryptone could be of clinical relevance for milk-drinking children infected with EAEC, as tryptone is a tryptic digest of the milk protein casein. 

The physiological conditions relating to EspC intoxication are also important for pathogenesis. Purified EspC is internalized by a non-specific pinocytic mechanism, does not bind to the plasma membrane at 4 °C (which indicates the absence of a specific surface receptor), and is weakly toxic in comparison to purified Pet [48]. In contrast, EspC was efficiently delivered to the host cell cytosol when cultured cells were exposed to EPEC [31,48]. EspC was released from EPEC by a type V secretion system and then entered the epithelial cells through the translocon of the EPEC type III secretion system [31]. EspC delivery to the host cytosol occurred after just 1 hour during EPEC-host cell interaction; this was much faster than the eight hours required for purified EspC to enter the cytosol of cultured cells incubated in the absence of EPEC. The EPEC-facilitated translocation of EspC could even occur *in trans*, as the combination of purified EspC and an EPEC ∆*espC* strain still resulted in rapid delivery of EspC to the target cell cytosol. Moreover, EPEC mutants with defective type III secretion systems were unable to translocate either endogenous or exogenous EspC into epithelial cells. This showed for the first time the efficient intracellular delivery of an autotransporter by the cooperative interactions between type V and type III secretion systems (Figure 1). The need for a type III secretion system to mediate EspC translocation into epithelial cells raises the question of how a milieu-secreted protein could gain access to the type III translocon for injection into host cells and suggests that the translocon apparatus is not a hermetic conduit from bacterial to eukaryotic cytoplasm.

Despite their structural similarities, Pet and EspC utilize distinct mechanisms to access the host cell cytosol. Endocytosed Pet follows an intracellular trafficking and translocation itinerary utilized by many AB toxins. In fact, Pet is the only known non-AB toxin to use retrograde trafficking and ERAD-mediated translocation to reach the cytosol. EspC also employs a unique translocation mechanism involving the coordinated action of type V and type III secretion systems. The pathogenesis of both toxins is affected by the physiological conditions of intoxication, and additional studies should further elucidate the influence of the pathogen itself on the intoxication process. 

## 6.  Effect of Pet and EspC on Fodrin and the Actin Cytoskeleton

After gaining access to the epithelial cytosol, both Pet and EspC target the actin-binding protein fodrin (also known as spectrin) [45,59]. However, Pet and EspC modify fodrin by different mechanisms.

Fodrin is an elongated heterodimer consisting of a 280 kDa α subunit and a 246 kDa β subunit [60,61]. Two heterodimers associate in a head-to-head orientation to form a functional tetramer. Both fodrin subunits contain multiple copies of a 106 amino acid motif termed the spectrin repeat as well as a src homology 3 domain, a pleckstrin homology domain, a calmodulin binding domain, and an actin-binding domain. Through these domains, the fodrin tetramer anchors membrane lipids and transmembrane proteins to the cortical actin cytoskeleton which lies beneath the plasma membrane. The interaction between fodrin and filamentous actin provides a degree of structural organization to the actin cytoskeleton which helps the cell withstand mechanical stress. Fodrin is also involved in epithelial morphogenesis [62] and, when cleaved in the 11th repetitive unit by calpain or caspase-3, apoptotic cell death [63,64]. 

Pet displays affinity for α-fodrin *in vitro* and cleaves epithelial fodrin *in vivo* [59,65]. Two breakdown products of 37 kDa and 72 kDa were generated from *in vitro* Pet activity against a recombinant GST-tagged construct that contained a 109 kDa fragment of α-fodrin representing the 8th to the 14th spectrin repeats (codons 809-1529) [59]. This was the first report showing cleavage of α-fodrin by a bacterial protease. Cleavage occurred between residues M^1198^ and V^1199^ within the calmodulin-binding domain of fodrin's 11th repetitive unit (Figure 1). Site-directed mutagenesis of these amino acids prevented GST-fodrin degradation by Pet. An inactivating I260S mutation in the Pet serine protease motif also prevented the proteolysis of GST-fodrin. 

Pet also cleaves epithelial fodrin in cultured cells [59,65]. *In vivo* proteolysis of fodrin did not occur in the presence of PMSF or with the Pet I260S mutant toxin. Pet activity against fodrin generated a 120 kDa breakdown product which was found in intracellular aggregates as membrane blebs. Loss of fodrin disrupted the structural link between the plasma membrane and the cortical actin cytoskeleton. Contraction of the actin cytoskeleton, loss of actin stress fibers, cell rounding, and eventual detachment from the substratum resulted from the loss of proper actin architecture. In intestinal epithelial cells, fodrin proteolysis may also lead to disassembly of the microvilli: the lower part of the actin filament bundle in the microvilli core is anchored and stabilized by a specialized structure which contains a dense core of fodrin [66]. Cleavage of α-fodrin to a 120 kDa fragment has been detected in apoptotic cells [63,64], so Pet activity may contribute to enterocyte death by triggering an apoptotic pathway. 

The ability to cleave epithelial fodrin and thereby alter the cytoskeleton of cultured cells is dependent on the integrity of the serine protease motif of Pet [59,65]. This is in good accordance with prior observations that the cytopathic effects of Pet are linked to its serine protease motif [32,46]. The collective data from *in vitro*, cell culture, and organ culture studies support a model in which Pet, by virtue of its serine protease activity, cleaves α-fodrin. This event inactivates fodrin and consequently leads to alterations of the actin cytoskeleton. Cell rounding, exfoliation, and disruptions to the integrity of the epithelial monolayer ensue. Pet may also contribute to pathogenesis by collapsing the microvilli of intoxicated cells and generating an apoptotic response. 

Like Pet, EspC cleaves GST-fodrin in a reaction that requires a functional serine protease motif [45]. However, unlike Pet, fodrin proteolysis by EspC generates four subproducts with apparent molecular masses of 72, 43, 45, and 34 kDa. The last two fragments come from further processing of an initial fodrin proteolytic fragment of around 72 kDa; suggesting two cleavage sites: fodrin's 11th and 9th repetitive units (Figure 1). The recognition of separate fodrin proteolytic sites by Pet and EspC was further emphasized by competition studies using the inactive Pet S260I and EspC S256I mutant toxins [45]. These non-functional toxins are able to enter epithelial cells but are unable to cleave fodrin or damage the actin cytoskeleton. An excess of EspC S256I blocked the cytoskeletal damage caused by wild-type EspC, but it did not block the cytoskeletal damage caused by Pet. Moreover, an excess of Pet S260I did not prevent the cytoskeletal damage caused by wild-type EspC. The inhibition of wild-type EspC by EspC S256I could not result from competition for surface binding sites, as purified EspC enters cultured cells by a pinocytic mechanism that does not involve a specific surface receptor [48]. Thus, the inhibition of wild-type EspC by EspC S256I was most likely due to competition for the fodrin binding/proteolysis site. The lack of competition between Pet and EspC consequently indicated that the two toxins bind to separate regions of fodrin. These data collectively demonstrated that Pet and EspC recognize different binding and cleavage sites in fodrin. 

The distinct patterns of fodrin proteolysis by Pet and EspC result in distinct cellular effects. Both toxins generate cytopathic and enterotoxic effects through disruption of the actin cytoskeleton. However, fodrin degradation by EspC is not accompanied by redistribution of the proteolytic fragments to membrane blebs [45]. Furthermore, the fodrin proteolytic fragments generated by EspC do not correspond to the 120 kDa breakdown product resulting from Pet activity against the calmodulin-binding domain of fodrin [59,65]. EspC cleavage sites occur outside of the calmodulin-bindng domain, although one cleavage site is close to this domain. Since calmodulin and calpain I coordinately regulate the interaction between fodrin and filamentous actin to maintain the cytoskeletal integrity [67], the fact that Pet but not EspC cleaves within the calmodulin-binding domain of fodrin is perhaps related to the different cellular effects elicited by the two toxins. The cleavage of fodrin by calcium-dependent proteases has also been observed in several cellular processes, and this proteolysis can lead to various necrotic and apoptotic events [68]. Calpain and caspase-3 both cleave fodrin at adjacent sites near the calmodulin-binding domain in the 11th repetitive unit to generate distinct 150 kDa breakdown subproducts: calpain cleaves between V^1176^ and G^1177^, while caspase-3 cleaves between D^1185^ and S^1186^. Calpain subsequently cleaves the fodrin proteolytic fragment again, between G^1230^ and S^1231^, to produce a slightly smaller breakdown subproduct of 145 kDa. In addition, caspase-3 cleaves fodrin within the 13th repetitive subunit, between D^1478^ and S^1479^, to produce the apoptosis-specific breakdown subproduct of 120 kDa [68]. In a similar manner, the differential cleavage of fodrin by Pet and EspC could trigger different biological events in addition to the shared disruption of filamentous actin.

It is clear that EspC and Pet have different activities on epithelial cells, and further studies are in progress to understand the links between EspC, fodrin proteolysis, and EPEC pathogenesis. Even though both EspC and Pet have the same serine protease motif belonging to the SPATE subfamily, it is clear that the overall conformation of the toxin can influence its specific interaction with the target protein. The differences between EspC and Pet suggest a specialized role for EspC in EPEC pathogenesis which may be different than the role of Pet in EAEC pathogenesis.

## 7. Summary

Pet and EspC are SPATE enterotoxins produced by diarrheagenic EAEC and EPEC, respectively. The two toxins are highly homologous and share the same fodrin target. Proteolysis of fodrin disrupts the organization of the actin cytoskeleton, so both toxins produce cytopathic and enterotoxic effects. Despite these similarities, Pet and EspC exhibit distinct interactions with the host cell. Pet is secreted into the medium, enters the target cell by receptor-mediated endocytosis, escapes the endomembrane system to enter the cytosol, and cleaves fodrin at a single site between residues M^1198^ and V^1199^. EspC is also initially secreted into the medium, but it is captured by EPEC and injected into the host cell cytosol through a type III secretion system. Cytosolic EspC then cleaves fodrin at two sites that are separate from the Pet proteolytic site. Continued work on Pet and EspC should help decipher how the different host-pathogen interactions for these two toxins contribute to the distinct pathogenesis of EAEC and EPEC.
